# Supplementary material for: COVID-19: Spatial analysis of hospital case-fatality rate in France
Source: PLoS One. 2020 Dec 15;15(12):e0243606. doi: 10.1371/journal.pone.0243606 (PMC7737987; doi:10.1371/journal.pone.0243606)
Supplement: Attachment — Submitted filename: Response to Reviewers_3.docx [file pone.0243606.s003.docx]

**Response to the reviewers**

PONE-D-20-17129 COVID-19

From: Dr. Marc Souris, Directeur de Recherche, UMR 190 « Unités des virus émergents »

Object: Rebuttal letter to PONE-D-20-17129 COVID-19 review untitled “Spatial Analysis of Hospital Case-Fatality Rate in France” by Marc Souris and Jean-Paul Gonzalez.

To PLOS ONE Editor

Dear Editor,

We thank the reviewer for the last comments on the revised version of our manuscript. You will find enclosed the revised version of the manuscript that addresses all the comments raised by the reviewer #4 (the only one that had comments). As requested, our re-submission includes the following items:

- **The present letter** as a rebuttal letter responding in detail to each point raised by the reviewer.
- **A marked-up copy of our manuscript** with all changes made to the original version (file ”Revised Manuscript_3_with Track Changes”).
- **A copy of our manuscript** using track changes as been uploaded as a *supporting information* file.
- **An unmarked version** of our revised paper without tracked changes (file “Revised Manuscript_3.docx”, clean copy of the edited manuscript uploaded as the new *manuscript* file)

Please find below our answers addressing the comments raised by the reviewer#4.

1) *The concrete results in the manuscript include spatial autocorrelation of certain variables, a positive correlation between hospitalization rate and case-fatality rate, and a “weak” negative correlation between hospitalization rate and severity rate. However, there is concrete data and rigorous analysis to support one point the authors tried to stress—non-comparability of case-fatality rates across countries. Therefore, statements on this point should at most be kept in Discussion and removed from Abstract (the 4th paragraph) and Introduction (the 4th paragraph).*

Answer: we removed the statement of the introduction (effectively it is a result of our study), but we think that it can be kept as a result in the abstract. We move the statement at the end of the abstract to clearly show it as a conclusion.

2) *The exact definition of age standardized case-fatality rate is still unclear. In the formula in page 4, are P[a] and T[a] district specific or not? If they are, there should be subscript i; if not, that means the expectation E is the same in all the district of the France, which is the so-called “average rate for France”? It is unclear whether the district observed case fatality rate was age adjusted or not? My understanding is yes according to the term “district’s SLR”; however, what’s the formula for it? As I pointed out, the only formula so far is not district specific.*

Answer: Indirect age standardization use case-fatality rates by age group a (T_a_) calculated over the whole country, and not district specific, but P_a,i_ is the population of age group a in the district i. The subscript *i* was missing for P_a,i_. We corrected this error, and we thank the reviewer to have highlighted this error in the formula.

3) *The language is verbose and many contents are superfluous. I suggest shortening the manuscript, removing unnecessary parts, and making only succinct points.* *Take an example, the whole page 4 seems can be removed.*

Answer: we agree that the manuscript is long, but we think that explanations on lethality and its different factors are required to better understand the article (as noted positively by the other reviewers).

4) *The last sentence of the second paragraph in Introduction—“…, unlike case-fatality rates, which are normally calculated independently of the number of infected persons”— it is confusing to me, how the case-fatality rate could be independent of the number of infected persons? Isn’t it the denominator?*

Answer: The sentence meaning is: the case-fatality rate is independent of the extend of the disease in the population. We removed the sentence to avoid confusion.

5) I confirm that all the data used in this study is fully available, as indicated in the article.

Dear Editor, we thank you for your kind consideration of our reviewed manuscript.

Dr. Marc Souris

Dr. Jean-Paul Gonzalez
