# Supplementary material for: COVID-19: Spatial analysis of hospital case-fatality rate in France
Source: PLoS One. 2020 Dec 15;15(12):e0243606. doi: 10.1371/journal.pone.0243606 (PMC7737987; doi:10.1371/journal.pone.0243606)
Supplement: Attachment — Submitted filename: Response to Reviewers_4.docx [file pone.0243606.s004.docx]

**Response to the reviewers**

PONE-D-20-17129 COVID-19

From: Dr. Marc Souris, Directeur de Recherche, UMR 190 « Unités des virus émergents »

Object: Rebuttal letter to PONE-D-20-17129 COVID-19 review untitled “Spatial Analysis of Hospital Case-Fatality Rate in France” by Marc Souris and Jean-Paul Gonzalez.

To PLOS ONE Editor

Dear Editor,

You will find enclosed the revised version of the manuscript that addresses the last comment raised by the reviewer #4 (the only one that had comments). As requested, our re-submission includes the following items:

- **The present letter** as a rebuttal letter responding in detail to each point raised by the reviewer.
- **A marked-up copy of our manuscript** with all changes made to the original version (file ”Revised Manuscript_4_with Track Changes”).
- **A copy of our manuscript** using track changes as been uploaded as a *supporting information* file.
- **An unmarked version** of our revised paper without tracked changes (file “Revised Manuscript_4.docx”, clean copy of the edited manuscript uploaded as the new *manuscript* file)

Please find below our answers addressing the comments raised by the reviewer#4.

1) *The authors currently put in the last sentence of Abstract “…, suggesting marked international inconsistency in case reporting and death ascertainment/coding”, which is exactly what I commented—it is an implication from the results, not the direct results. Note that it is a very strong statement. Therefore, please remove it from Abstract and leave it only in Discussion.*

Answer: we removed the statement from the abstract.

Dear Editor, we thank you for your kind consideration of our reviewed manuscript.

Dr. Marc Souris

Dr. Jean-Paul Gonzalez
